# Transient Grating Spectroscopy in Magnetic Thin Films: Simultaneous Detection of Elastic and Magnetic Dynamics

**DOI:** 10.1038/srep29143

**Published:** 2016-07-05

**Authors:** J. Janušonis, T. Jansma, C. L. Chang, Q. Liu, A. Gatilova, A. M. Lomonosov, V. Shalagatskyi, T. Pezeril, V. V. Temnov, R. I. Tobey

**Affiliations:** 1 Zernike Institute for Advanced Materials, University of Groningen, Groningen, The Netherlands; 2 LAUM CNRS 6613, Université du Maine, 72085 Le Mans cedex, France; 3 IMMM CNRS 6283, Université du Maine, 72085 Le Mans cedex, France; 4 Fritz-Haber-Institut der Max-Planck-Gesellschaft, Abteilung Physikalische Chemie, Faradayweg 4-6, 14195 Berlin, Germany

## Abstract

Surface magnetoelastic waves are coupled elastic and magnetic excitations that propagate along the surface of a magnetic material. Ultrafast optical techniques allow for a non-contact excitation and detection scheme while providing the ability to measure both elastic and magnetic components individually. Here we describe a simple setup suitable for excitation and time resolved measurements of high frequency magnetoelastic waves, which is based on the transient grating technique. The elastic dynamics are measured by diffracting a probe laser pulse from the long-wavelength spatially periodic structural deformation. Simultaneously, a magnetooptical measurement, either Faraday or Kerr effect, is sensitive to the out-of-plane magnetization component. The correspondence in the response of the two channels probes the resonant interaction between the two degrees of freedom and reveals their intimate coupling. Unraveling the observed dynamics requires a detailed understanding of the spatio-temporal evolution of temperature, magnetization and thermo-elastic strain in the ferromagnet. Numerical solution of thermal diffusion in two dimensions provides the basis on which to understand the sensitivity in the magnetooptic detection.

Ultrafast phenomena in magnetic systems has been an active research topic for several decades^1^,^2^,^3^. In the context of applications, the ability to control magnetization on short timescales is important for the realization of fast magnetic memory and low power magnetic logic^4^,^5^. Many different mechanisms of magnetization dynamics activation have been explored - spin transfer torque^6^,^7^, thermal effects of the optical excitation^8^, nonthermal coupling and lately inverse magnetostriction. This latter aspect engages the material structure and its deformations to control magnetization dynamics. In particular, the bidirectional deformation of the lattice, i.e. compression or dilation, can lead to bidirectional control of magnetization, or, as shown in a number of recent reports, the control over precessional direction and amplitude^9^,^10^. In the high frequency limit, gigahertz acoustic waves coherently drive magnetization dynamics.

In order to investigate these high frequency, dynamic, magnetoelastic effects, one requires the ability to excite and monitor structural deformations while simultaneously monitoring the magnetization in a time resolved manner. One approach is to use interdigitated transducers to generate narrowband elastic waves^9^,^11^,^12^. In this case a harmonic voltage is applied to an interdigitated set of contacts evaporated on a piezoelectric substrate. The applied voltage leads to local substrate strains and their interference - to the excitation of planar elastic waves. The excitation is both wavelength and frequency selective where the contact spacing determines the wavelength and the radio frequency voltage determines the frequency. Achievable frequencies in the interdigitated contact approach are generally below 2 GHz and strain values cited in the literature are small^9^ - of an order 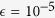.

A complementary approach relies on ultrafast laser excitation of a material, where thermoelastic processes are able to drive large amplitude strain waves that emanate from the excitation region. For example, in a “tight-focus” geometry, elastic waves propagate uniformly away from the focal spot, and have been shown to couple to the magnetization of the material^13^,^14^. Similarly, relaxing the tight focusing requirement, broadband longitudinal wavepackets propagating into the material bulk have also been shown to drive magnetization dynamics^10^,^15^,^16^,^17^.

Recently, we demonstrated a novel manner for generating surface magnetoelastic waves^18^,^19^, implemented using the ultrafast optical transient grating technique. This approach harnesses the efficiency and ease of optical spectroscopy (non-contact, easily adjustable, large amplitude strain generation) with the planar geometry associated with transducers. The transient grating (TG) geometry is a special configuration of time-resolved pump-probe spectroscopy in which two coincident pump pulses interfere at the sample surface to generate a spatially periodic excitation profile. The spatial periodicity is encoded onto the material properties, (e.g. carrier concentration, temperature, surface deformation) from which a time delayed probe beam can be diffracted to provide information about dynamics at a specific spatial length scale. TG has a long history for measuring dynamics of carriers at metal, semiconductor^20^,^21^, and superconductor^22^ surfaces, lateral nucleation dynamics in insulator-to-metal transitions^23^, as well as a range of measurements of elastic dynamics at surfaces of thin films and bulk materials^24^,^25^,^26^,^27^,^28^,^29^,^30^,^31^. TG spectroscopy continues to be developed in novel ways, including implementations of X-rays for detection of dynamics^23^,^25^, as well as recent demonstrations of X-ray generation of transient gratings^32^.

In this contribution we discuss the optical setup which extends the transient grating technique to incorporate magnetization sensitivity based on magnetooptic detection^18^,^19^. As we will show, transient diffraction enables us to follow structural dynamics (a feature already well-recognized^24^), while time resolved magnetooptics (Faraday rotation/ellipticity, Kerr rotation) measures the average magnetization dynamics (also a well recognized feature^33^). The novelty of our implementation is the demonstration of the intimate coupling between the two degrees of freedom, and furthermore the resonant interaction between the two under appropriate conditions of external applied magnetic field. In our experimental setup three time-resolved measurements can be performed simultaneously - 1) diffraction in transmission, 2) diffraction in reflection and 3) polarization analysis in transmission (cf. Fig. 1a,b) - the combination of which demonstrates the resonant interaction between elastic and magnetic degrees of freedom. Finally, we discuss the implications of the magnetooptics detection when performed on the periodic thermal background inherent to our geometry. As we will show, this feature leads to wave vector selective sensitivity and introduces a correction to the measured magnetization precession angles.

## Experimental Setup

At the front end of our experiment, pulses of light are generated by an amplified Ti:Sapphire laser system (Coherent Legend Elite) providing pulses of 120 fs duration at 1 kHz repetition rate and 5 mJ pulse energy. A portion of the laser output is directed to the experiment and split into pump and probe beams. The pump beam is directed onto a delay stage providing up to eight nanoseconds of time delay (initial measurements only provided 4 nanoseconds). The pump is frequency doubled to *λ* = 400 nm, while the probe light is kept at the laser fundamental *λ* = 800 nm. The pump beam is split in two and combined at the sample surface to form an intensity grating pattern to launch elastic waves. The sample is placed in a magnetic field which can be tuned between ±2000 G. The magnetic field can be rotated around the surface normal (magnetic field always in the plane of the sample).

**Figure 1:**
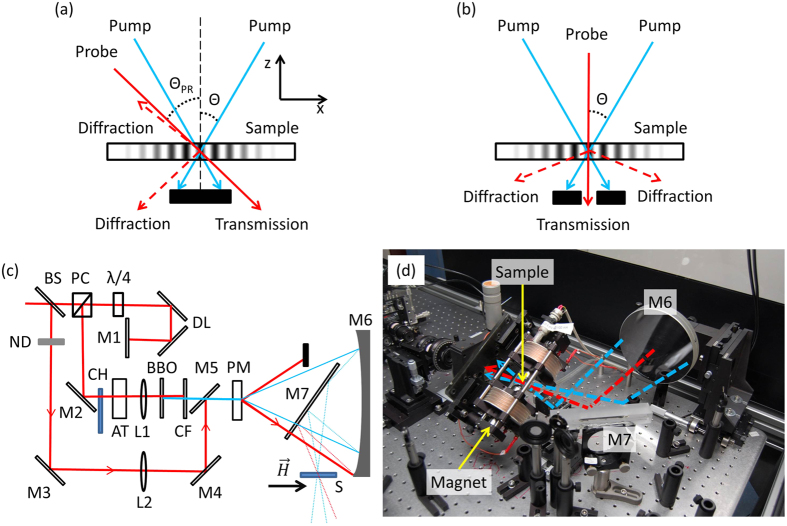
Optical arrangement at the sample position. Two linearly polarized 400 nm pump pulses overlap onto the sample at an angle 2*θ*, creating a sinusoidal intensity grating. (**a**) 800 nm probe pulses impinge at an angle *θ*_*PR*_. Probe light is simultaneously diffracted (both in transmission and reflection) and transmitted. The polarization dynamics of the transmitted probe (Faraday configuration) are analyzed to provide information on the average magnetization of the sample. Simultaneous measurement of diffraction provides information on the structural dynamics associated with the elastic waves. In this “Bragg configuration”, the smallest spatial periodicity is determined by the long wavelength probe and the size of upstream optical components (for us 2.2 *μ*m). (**b**) In a second configuration, probing is implemented at normal incidence, where now the smallest achievable spatial period is dictated by the shorter pump wavelength and the size of upstream optical components (for us 1.1 *μ*m). (**c**) Setup schematic in (**a**) configuration: BS - beamsplitter, PC - polarization cube, *λ*/4 - waveplate, DL - delay line, M1-M7 - mirrors, ND - neutral density filter, CH - chopper, AT - attenuator of the pump power, BBO - BBO crystal for second harmonic generation, CF - shortpass color filter to block fundamental beam, L1-2 focusing lenses, PM - phase mask, S - sample, 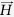 - magnetic field, applied in a sample plane. (**d**) The setup photo in (**b**) configuration: the focusing mirror M6, the folding mirror M7, compact rotatable magnet, and the sample holder are visible and pump (blue) and probe (red) beams are drawn.

Implementation of the transient grating is accomplished by spatially and temporally overlapping two pump pulses on the sample surface. An elegant manner in which to implement this is to image a binary phase mask (square profile phase grating) onto the sample surface^34^,^35^,^36^ (Fig. 1c). When the imaging condition uses only the first order diffraction maxima (±1) of the phase mask, a sine spatial profile is generated at the image plane. The imaging condition provides full fringe visibility across the entire spatial aperture of the beam (due to pulse front tilting associated with diffractive optics), while simultaneously simplifying the requirements for temporal overlap of the two pulses. This experimental geometry greatly simplifies the probe beam alignment when it is propagated through the same phase mask. In the so-called Bragg configuration, a single phase mask splits both the pump and probe beams to ensure that the probe impinges onto the pump-induced transient grating at an angle such that light is perfectly back diffracted along the incoming path, providing for easy location of both reflected and transmitted diffraction beams. Furthermore, heterodyne detection can be implemented with the aid of the secondary local oscillator. When probing with longer wavelength light, the Bragg configuration (Fig. 1a) limits the range of allowed grating periodicities due to the numerical aperture of the upstream optics which image the probe onto a sample. In our experiments, the Bragg configuration is limited to grating periods of 2.2 *μ*m. An alternative geometry we employ is that of normal incidence probing as shown in Fig. 1b. Here, both Faraday rotation and diffraction can be implemented, however alignment of the diffraction probes proves more challenging. The minimum grating periodicity probed is determined by the pump wavelength and numerical aperture of the upstream optics and extends down to 1.1 *μ*m.

In our implementation, pump and probe light are directed onto the phase mask, a precision etched quartz glass (Toppan Photomasks) providing for maximum 1^*st*^ order diffraction efficiency at the pump wavelength. A number of phase masks are etched onto a single glass substrate with periodicities ranging from 1.5 *μ*m to 18 *μ*m. The excitation periodicity can be changed by a lateral shift of the phase mask and a dovetail rail is installed for easy and repeatable phase mask movement. The phase mask is imaged onto the sample surface using only the two first order diffraction maxima while all other diffraction orders are blocked. The excitation periodicity on the sample surface is given by the phase mask periodicity, d, and the imaging magnification, 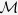: 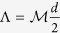.

Magneto-optics and diffraction detection are implemented using silicon photodiodes and lockin amplifiers, and where possible all detection channels are collected simultaneously. Magnetic dynamics in the Faraday or Kerr geometries are monitored via balanced detection of the induced rotation and ellipticity of the probe beam polarization^33^. The magnetooptic signals are proportional to the magnetization component along the probe wave vector 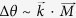, and thus the setup is sensitive to the magnetization as precession brings a component of the magnetization out of the sample plane. In the normal-incidence configuration (Fig. 1b), we are sensitive only to the out-of-plane component, while in the angled configuration (Fig. 1a) a component of the in-plane magnetization direction might also affect the signal. For small precession angles *α*, this component varies like *α*^2^ and is negligible. In our measurements to date, both detection schemes provide for qualitatively similar results. Regardless of grating periodicity or chosen detection geometry, dynamics in either the Faraday or Kerr configurations can be realized, however, for partially transparent thin films Faraday configuration is simpler to implement.

## Measurement Example

In order to illustrate the capabilities of the setup we discuss a sample dataset, measured on a 40 nm thick nickel film on a 1 mm thick soda lime glass substrate (microscope slide). The sample was excited with 2.4 *μ*m periodicity, implemented in the Bragg experimental geometry as shown in Fig. 1a, and simultaneous acquisition of both transmission and reflection diffraction as well as magnetooptics was enabled. The equilibrium magnetization was in the plane of the sample and at a small angle with the grating wave vector 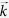. The sample was initially poled with *H* ≈ 2000 G and then measurements were performed at zero field.

In opaque (or partially opaque) materials such as metallic thin films, transient grating excitation results in the thermoelastic generation of acoustic waves, where the spatially periodic excitation locally heats the lattice and results in a spatially periodic stress profile. The experimental geometry is wavevector selective, and thus all elastic waves (at any frequency) can be excited subject initial conditions of the thermal stress. We will show that two dominant surface propagating elastic waves are generated in this geometry: the Rayleigh Surface Acoustic Wave (SAW) and the Surface Skimming Longitudinal Waves (SSLW). In the former case, the penetration depth of the elastic deformation is roughly equal to the excitation wavelength^37^, Λ, where in our measurements 1.1 *μ*m < Λ < 8 *μ*m. As the majority of elastic energy is contained within the substrate material, the propagation velocity is largely determined by the substrate properties, and exclusively so when Λ is much greater than the film thickness. In the case of SSLW, the elastic wave is ‘leaky’, and energy is quickly carried away from the surface region.

**Figure 2:**
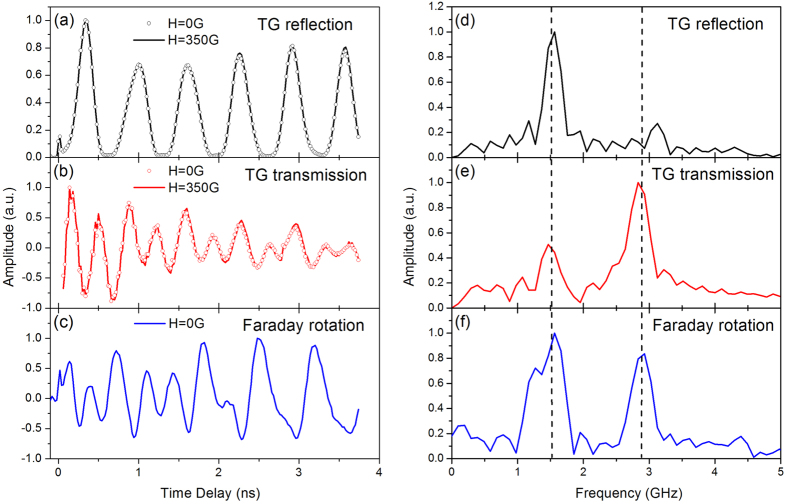
40 nm nickel film on soda lime glass, excited with a grating periodicity of 2.1 *μ*m. Transient grating diffraction in reflection (**a**) and transmission (**b**) at two applied fields shows no sensitivity to magnetic field. (**c**) Faraday rotation signal at zero magnetic field with field dependence shown in Fig. 3. Fourier Transforms of (**a–c**) are shown in panels (**d–f**) respectively, demonstrating that the Faraday response is sensitive to both elastic waves while the two diffraction channels exhibit different sensitivities to elastic transients. Transient diffraction in reflection exclusively measures the surface corrugation due to SAW, while in transmission a combination of surface corrugation and photoelasticity leads to sensitivity of both SAW and SSLW.

Diffraction in the two configurations (reflection and transmission) are shown in Fig. 2(a,b) where differences in sensitivity can readily be seen. The reflection channel exhibits low frequency oscillations with minimal attenuation in our time window. Meanwhile, the transmission channel exhibits high frequency, small amplitude oscillations with large attenuation. We understand these differences to arise from differing sensitivities in the two detection channels. For the case of reflection diffraction, light is diffracted due to periodic phase variation imposed by surface deformation, and is less sensitive to photoelastic effects in the film. Surface acoustic waves such as the Rayleigh mode are known to have large surface displacements, and thus lead to large amplitude diffraction. Conversely, the transmission geometry case is more sensitive to the phase variation arising from photoelastic effects. Here the probe light propagates through a film/substrate heterostructure that is significantly longer than *λ*_*probe*_ and thus phase accumulation over the propagation length must be included. The difference in measured frequencies is displayed as a Fourier Transform (FT) of the signal in Fig. 2(d,e) to illustrate the differences in detection sensitivity.

The new aspect that we implement in these measurements is the additional magneto-optics measurements subject to TG excitation. The results of this detection channel are shown in Fig. 2(c), where a complex series of oscillations can be seen. As in pump-probe spectroscopy, we understand that the Faraday analysis of the transmitted beam measures the *average* magnetization dynamics of the sample. In the parlance of transient gratings, no lateral momentum is provided by the spatially periodic excitation and thus we measure the zero spatial frequency response (the average value). Understanding the response of Fig. 2(c) is aided by the Fourier Transform, which is shown in Fig. 2(f). Comparing the FT to those of the elastic signals, it is now clear the oscillations observed in the Faraday detection channel match exactly those frequencies observed in the two diffraction channels. This result is therefore suggestive of the intimate coupling between the elastic and magnetic degrees of freedom in this material system^18^,^19^.

**Figure 3:**
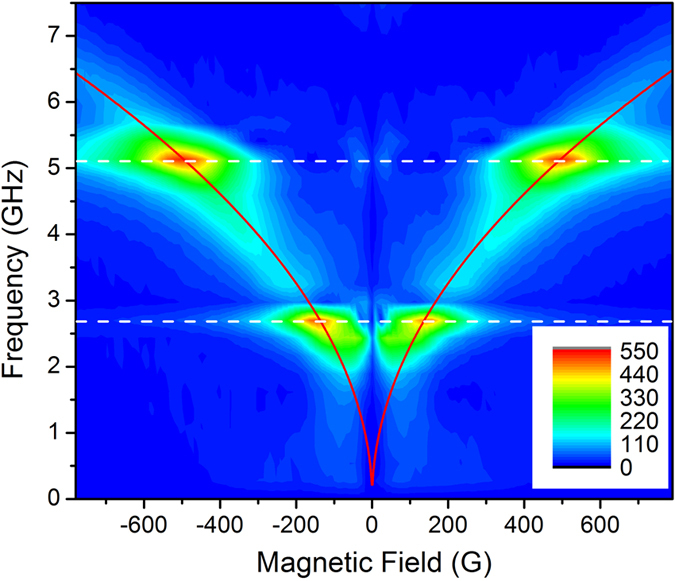
The magnetization precessional amplitude strongly depends on the field applied to the sample. When the field tuned ferromagnetic resonance frequency (red) matches that of the elastic waves (white), large amplitude oscillations are observed. The elastic frequencies can be determined by the transient grating measurements. Figure reproduced from Janusonis *et al*.^19^.

Next, in order to demonstrate that the Faraday detection is solely sensitive to the magnetization dynamics in the film (the substrate is not magnetic), we perform a magnetic field scan, which we show in Fig. 3 (reproduced from Janusonis *et al*.^19^) and displays the amplitude of Faraday oscillation as a function of applied field. Magnetic precession is demonstrated using experimental configuration in Fig. 1b on a grating periodicity of 1.1 *μ*m. In the figure, the elastic waves are indicated by white horizontal lines and the field-tuned ferromagnetic resonance is shown in red. From the data plotted in Fig. 3, it is clear that at the intersection of elastic and FMR frequencies the precession amplitude is largest. We also perform magnetic field dependencies of the transient diffraction signals and show that they are insensitive to an applied magnetic field. Data for the reflection and transmission channels are overlaid onto the curves in Fig. 2(a,b). This is an expected result since diffraction in the TG configuration has been implemented for several years to measure elastic dynamics for magnetic thin films, and nickel in particular^25^,^26^, with no sensitivity to magnetization dynamics. Apparent discrepancies between the data shown in Figs 2 and 3 arise from the details of the magnetic field alignment for the two data sets. As the angle between applied field and acoustic wavevector is varied, the amplitude at resonance and the near-zero-field Faraday behavior changes (and will be the subject of a forthcoming report). The data displayed in Fig. 2 was taken with an angle of 7.5° between 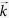 and 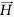, while the data shown in Fig. 3 below was taken at an angle of 60°. The clear difference between these two angles is the appearance of oscillation amplitude at low applie fields when the angle is small. However, the general features of resonant interaction, i.e. the amplification of precessional motion due to the elastic driving field, are unaffected.

## Magnetooptic Sensitivity

The magnetic sensitivity in the Faraday channel relies on a non-trivial interplay between elastically activated magnetostatic spin waves and the optically induced thermal gradieynt along its period. Over one period of the elastic wave, the magnetization precession samples all phases of oscillation and therefore the detectable net out-of-plane magnetization of the sample would sum to zero. It is additional aspect of thermal excitation that suppresses a portion of this magnetostatic wave, and provides for a net magnetization that is measured in the Faraday configuration. Thus, the spatial profile of the magnetic wave as well as the thermal profile, and its dynamics, dictate the measurement sensitivity.

The magnetic waves are excited by the underlying elastic waves via inverse magnetostriction. In the TG configuration, two counterpropagating elastic waves are generated for both SAW and SSLW. The effects we describe can equally be accounted for by considering the time dependent elastic distortion as a standing wave, with regions of both positive and negative in-plane strain. In either picture, the strain profile results in a spatial distribution of the out-of-plane magnetization component:


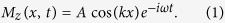


Equation 1 describes a spatially non-uniform magnetization profile, where the out of plane component gradually changes as function of position. In the elastic standing wave picture, we note that nodes in the elastic wave strain (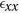) provide no torque to the magnetization, and thus these are regions of space where the magnetization is stationary to a first approximation. On either side of the nodes, magnetization precession is *π* out of phase.

In addition to this dipolar magnetostatic wave, the TG excitation also generates a spatially periodic thermal profile *T*(*x*). Saturation magnetization *M*_*S*_(*T*) at an elevated temperature decreases with respect to the saturation magnetization at zero temperature *M*_*S*_(0) as described by the Curie-Weiss law. Thus the temperature profile modifies the spatial amplitude of the magnetization. The combined effect of the magnetostatic wave and the temperature profile can be written for out of plane component as:


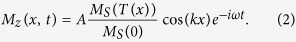


The net out-of-plane magnetic vector will be an average of this expression over one full spatial period


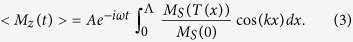


Equation 3 relates the measured result <*M*_*z*_> with the ‘real’ precession amplitude of the propagating magnetic wave *A*, so this expression can be also called measurement sensitivity. This correction is necessary in order to estimate absolute precession amplitude and its dynamics.

The measured magnetization is therefore determined by the details of the temperature dependence of the magnetization profile, *M*_*S*_(*T*(*x*)), and its phase relative to the envelope of a standing magnetic wave, cos(*kx*). While magnetization profile can be calculated from the temperature distribution based on a Curie-Weiss law or experimental data, the phase of the magnetic wave relative to the thermal background can be determined considering the strain/displacement relation. The thermal profile is symmetric around the maximal and minimal temperature points, those points experience zero displacement (along x) and therefore maximum strain amplitudes.

**Figure 4:**
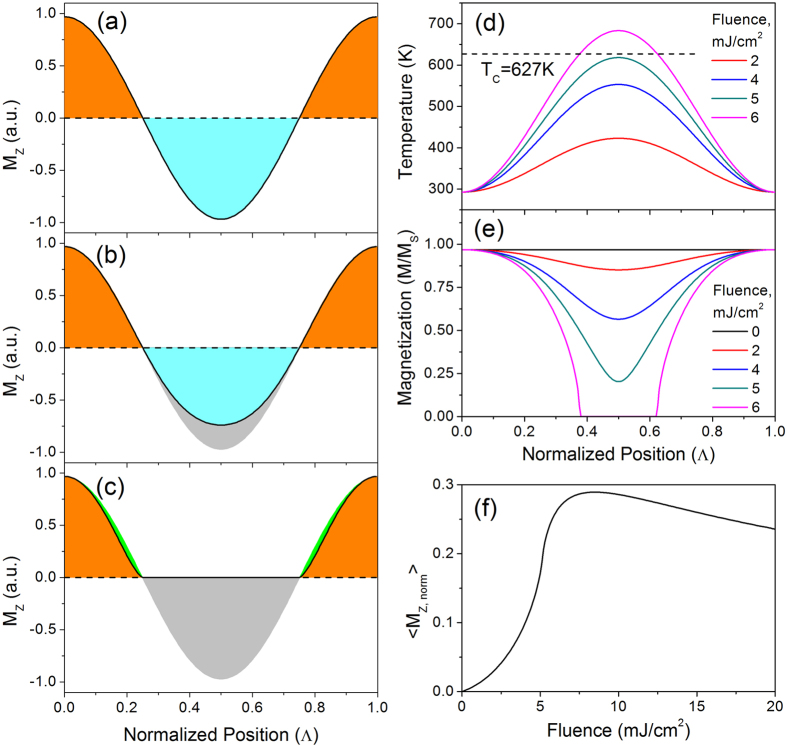
Magnetic wave detection on a periodically heated background. (**a**) A long wavelength magnetostatic spin wave samples all phases of precession orientation and thus the out-of-plane component, *M*_*z*_, averages to zero over a the full period. (**b**) A spatially periodic thermal excitation suppresses the magnetization, and correspondingly *M*_*z*_. Under low fluence conditions, one phase of precession is suppressed resulting in a net magnetization. (**c**) Under high fluence conditions, more than half of the period demagnetizes. (**d**) Temperature profiles after the equilibration throughout the thickness of the 40 nm thick film (corresponding to t ≈ 35 ps after the excitation) and corresponding magnetization values (**e**), normalized to the saturation value at T = 0 K. Due to the spatially dependent suppression, the detected magnetization is a fraction of the magnetostatic wave amplitude. The sensitivity dependence on fluence is shown in (**f**).

These effects are pictorially represented in Fig. 4(a–c) as the M_*z*_ component of the precession amplitude. In the absence of a thermal gradient (Fig. 4(a)), equal values of positive and negative M_*z*_ values appear over one period of elastic deformation, providing a net zero magnetic moment. However, when the thermal profile is taken into account (here the hottest portion of the sample is at the midpoint of the displayed period), one phase of oscillation is suppressed, and thus a net magnetization emerges. The temperature and magnetization profiles for a 40 nm nickel film are plotted in Fig. 4(d,e) respectively for a range of excitation fluences (and for one point in time, ≈35 ps, when the temperature is equilibrated across the thickness of the film but lateral diffusion is still negligible). As the fluence is increased, the thermally excited region continuously reduces its magnetization and at some value of fluence the magnetization is completely suppressed. The measured net moment is largest when approximately half of the period is above Curie temperature, whereas if the fluence is increased further, other portions of the period begin to demagnetize (Fig. 4(c)) and the averaged magnetic moment decreases. This effect is summarized in Fig. 4(f) by depicting the ratio between the net magnetic moment and the amplitude of the magnetic wave. Thus we are left with calculating the thermal profile in the TG configuration, and the corresponding magnetization profile.

The temperature and magnetization dynamics are calculated using the two-temperature model to assess the initial temperature distribution and then subsequently the COMSOL multiphysics simulation package to include lateral and longitudinal thermalization^38^. For the thin film on substrate heterostructure, thermal diffusion into the substrate includes the possibility of thermal boundary resistance (Kapitza resistance) which is extracted from single beam excitation pump probe data (no TG) and is estimated to be less than 10^−8^ Km^2^/W. In simulations we further varied the values of Kapitza resistance in the range 10^−9^–10^−8^ Km^2^/W and didn’t observe any significant deviations in temperature dynamics. We ascribe this effect to the low thermal conductivity of the glass substrate (k = 0.9 W/Km), which largely determines the speed of thermal transport from the hot nickel layer to the cold substrate.

**Figure 5:**
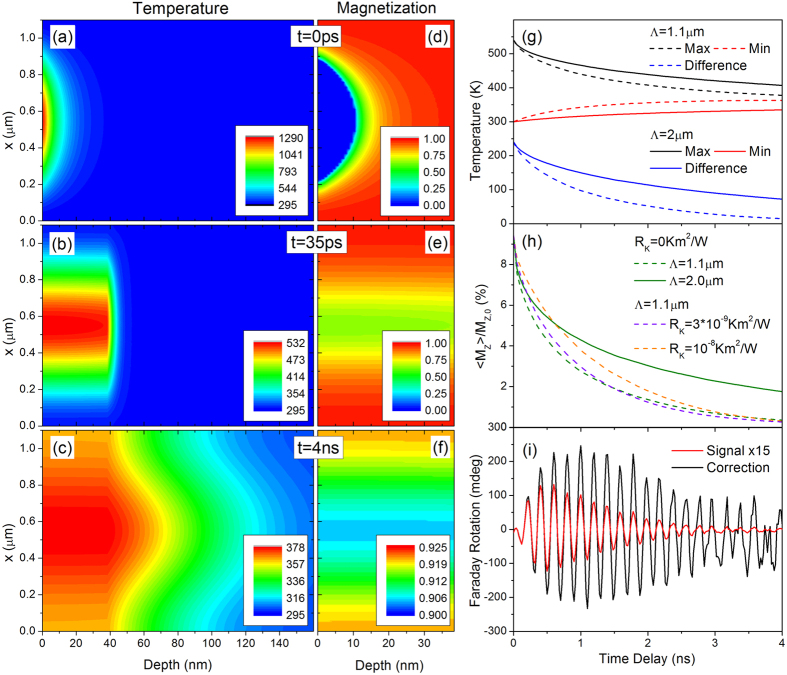
Calculation of the time dependent magnetic contrast requires a detailed knowledge of the temperature evolution and the resulting magnetization amplitude. Panels (**a–c**) show the temperature profiles at three different time slices, t = 0 ps, 35 ps, and 4 ns. Calculation of the initial dynamics is based on the two-temperature model, while subsequent thermalization is calculated using the COMSOL simulation package. The time axis starts at the moment of the electron-lattice thermalization, typically <2 ps after the pump pulse, with excitation fluence of 3.6 mJ/cm^2^. Note that the vertical length scale is in microns while the depth length scale is in nanometers. Panels (**d–f**) show the spatial distribution of the magnetization, where unity represents the magnetization at zero temperature. The dynamics of the hottest and coldest points of the sample are shown in panel (**g**) for grating periodicities of 1.1 *μ*m and 2 *μ*m, along with the difference in temperature. Panel (**h**) displays the resultant contrast which incorporates both the precession amplitude and temperature profile. Also shown are the different contrast curves which incorporate variations in the thermal boundary resistance between film and substrate. Finally in (i) the Faraday measurement resonant with the SSLW excitation is corrected for the magnetic contrast.

Solutions to the temperature and magnetization profiles at a modest excitation fluence 3.6 mJ/cm^2^ are included in Fig. 5(a–c), for time slices 0 ps, 35 ps, and 4 ns to provide a range of sample temperatures experienced in our measurements. Note that in the simulation, the lateral lengthscale is 1.1 *μ*m while into the sample depth we only plot to 100 nm (sample thickness 40 nm) and therefore the spatial temperature gradients along the surface normal appear to be much larger. In the plots, one sees a fast thermalization across the entire film thickness by 35 ps, while lateral thermalization and thermalization into the substrate can be seen on 4 ns delays. Simultaneously, we show the depth profile of the sample magnetization in Fig. 5(d–f) for the same time slices.

Figure 5(g) shows the time dynamics of the temperature at the hottest and coldest points of the film with the temperature difference plotted below. The magnetization contrast is then an integration over the M_*z*_ component over one full period of the strain/temperature modulation over the thickness of the nickel film. A plot of the time evolution of the magnetic contrast for the pump fluence of 3.6 mJ/cm^2^ for both Λ = 1.1 *μ*m and Λ = 2.0 *μ*m is shown in Fig. 5(h). As expected, thermalization effects limit the Faraday visibility more strongly when the grating period is smaller, suppressing the magnetic sensitivity at shorter time delays. These plots also demonstrate the effect of varying the Kapitza resistance, which impacts thermalization across the film/substrate interface, and ultimately on the magnetic sensitivity.

The primary consequence of this distribution of magnetization angles and magnitudes is that the acquired data must be corrected by this time dependent curve in order to extract the true precessional motion of the magnetic wave. Thus, as final step, we take the data collected at a magnetic field H = 500 G (corresponding to the SSLW acoustic wave in resonance with the magnetic precession) with the Λ = 1.1 *μ*m and divide it by the magnetization contrast to reveal the actual precessional dynamics of the magnetization which we display in Fig. 5(i). Here we now see that the correction increases the precessional amplitude and also changes the dynamic profile. The precessional motion remains a complex process, especially for the case of resonance with SSLW. The observed buildup and attenuation of magnetization precession depends on the resonant interaction between elastic and magnetic modes, the attenuation of the leaky elastic wave, and finally the magnetic sensitivity that we have detailed above. Since the elastic wave is also being measured separately (and if it were measured for long enough in time) the magnetic precession could be fully determined over the course of many tens of nanoseconds and beyond.

## Conclusions and Outlook

We have presented a simple optical spectroscopy setup for measuring magnetoelastic waves in thin magnetic samples. Our technique is based on the well-known transient grating setup which we have augmented to detect diffraction and magnetooptical dynamics simultaneously. By detecting diffracted light in reflection and transmission geometries, we are able to address different aspects of the structural dynamics while the simultaneously acquired Faraday rotation measures the magnetic dynamics. The correspondence between all channels allows us to uniquely identify the intimate interaction between coupled elastic and magnetic degrees of freedom. Applying an in-plane magnetic field results in the resonant coupling between elastic and magnetic components, namely, when the elastic frequency matches that of the ferromagnetic resonance, a sharp increase in magnetic precession amplitude is witnessed.

We understand the measured precessional dynamics to arise from a combination of elastic driving force, and resonant coupling, as well as a sensitivity function that accounts for the temperature dependent magnetization as a function of time. The spatially periodic temperature dynamics are described using a two-temperature model and thermalization modeling using COMSOL, while the temperature dependent magnetization is extracted from a Curie-Weiss law for Nickel. This combined approach allows us to extract a time dependent sensitivity, which we understand as a scaling factor between the observed magnetization amplitude and the actual precessional amplitude.

We foresee a number of interesting applications of our technique, particularly the use of magnetoelastic probing of deeply subwavelength transient gratings. New capabilities in free electron laser science have made possible X-ray generation of transient gratings^32^ with grating periods of tens of nanometers and below. Conventional transient grating spectroscopy requires the diffraction of a short wavelength probe that satisfies the Bragg diffraction condition, implying that X-ray probes are required to diffract from X-ray induced transient gratings.

One of the consequences of detecting the *average* magnetization using a normal incidence probe is that we have opened a new detection channel to dynamics occurring at a fixed wavevector, while the dynamics are encoded onto an average material properties. In our measurements to date, we have demonstrated that elastic and magnetic degrees of freedom are intimately coupled and that the oscillation frequency measured in the magnetic degree of freedom is equal to that of the elastic degree of freedom. Thus elastic dispersion can be probed without a diffracted probe beam. Furthermore, owing to the complex elastic profiles that are generated as the grating periodicities begin to coincide with film thickness (the waveguided modes of thin films), magnetoelastic coupling could begin to excite and probe the interactions between narrow band elastic modes and *exchange coupled* magnons. Here the specific choice of grating period, material, and applied field can be used to tune the dispersion relations of both magnon (quantized along the film thickness or in plane in the limit of very small gratings) and the elastic modes to effectively couple the two modes and provide for a new coherent control methodology where the material structure, and it’s deformations, can be used to coherently excited quantized magnetic modes in materials.

The presented technique may also be helpful to provide the direct acces to the fundamental magneto-acoustic non-reciprocity effects. Indeed, the coupling between the magnetic and elastic degrees of freedom leads to formation of magneto-elastic surface (eigen)modes with propagation velocities depending on the direction of the external magnetic field and sometimes different for opposite orientations of the external magnetic field. This magneto-elastic non-reciprocity effect is proportional to the magnitude of the magnetostriction coefficient and is quite small for the case of transition metals. However, it can be boosted in giant magneto-strictive materials like Terfenol-D^15^ and we are looking forward to probing the magneto-elastic interactions in functional materials with optimized properties, which would also enable the excitation of large-angle magnetization precession ultimately leading to the nonlinear magnetization dynamics.

## Additional Information

**How to cite this article**: Janušonis, J. *et al*. Transient Grating Spectroscopy in Magnetic Thin Films: Simultaneous Detection of Elastic and Magnetic Dynamics. *Sci. Rep.* **6**, 29143; doi: 10.1038/srep29143 (2016).
